# Persistent anomalies of the extratropical Northern Hemisphere wintertime circulation as an initiator of El Niño/Southern Oscillation events

**DOI:** 10.1038/s41598-017-09580-9

**Published:** 2017-08-31

**Authors:** Bruce T. Anderson, Pedram Hassanzadeh, Rodrigo Caballero

**Affiliations:** 1 Department of Earth and Environment, Boston University, 685 Commonwealth Ave., Boston, MA 02215 USA; 2 Departments of Mechanical Engineering and Earth Science, Rice University, 6100 Main St., Houston, TX 77005 USA; 3 Department of Meteorology and Bolin Center for Climate Research, Stockholm University, 106 91 Stockholm, Sweden

## Abstract

Climates across both hemispheres are strongly influenced by tropical Pacific variability associated with the El Niño/Southern Oscillation (ENSO). Conversely, extratropical variability also can affect the tropics. In particular, seasonal-mean alterations of near-surface winds associated with the North Pacific Oscillation (NPO) serve as a significant extratropical forcing agent of ENSO. However, it is still unclear what dynamical processes give rise to year-to-year shifts in these long-lived NPO anomalies. Here we show that intraseasonal variability in boreal winter pressure patterns over the Central North Pacific (CNP) imparts a significant signature upon the seasonal-mean circulations characteristic of the NPO. Further we show that the seasonal-mean signature results in part from year-to-year variations in persistent, quasi-stationary low-pressure intrusions into the subtropics of the CNP, accompanied by the establishment of persistent, quasi-stationary high-pressure anomalies over high latitudes of the CNP. Overall, we find that the frequency of these persistent extratropical anomalies (PEAs) during a given winter serves as a key modulator of intraseasonal variability in extratropical North Pacific circulations and, through their influence on the seasonal-mean circulations in and around the southern lobe of the NPO, the state of the equatorial Pacific 9–12 months later.

## Introduction

The near-surface characteristics that determine a region’s climate are strongly related to large-scale processes occurring tens of thousands of meters up in the atmosphere and spanning thousands of kilometers^1^. Variations in the atmospheric circulations at these altitudes and on these scales can be generated by slowly-evolving changes of the equatorial Pacific associated with the El Niño/Southern Oscillation (ENSO)^2–4^, which produces hemispheric-scale circulation changes in both the tropics and extratropics^5–7^. In addition, though, seasonal-mean variations in extratropical circulations can be sustained by the repeated occurrence of persistent (longer-than-synoptic) quasi-stationary extratropical anomalies (hereafter persistent extratropical anomalies or PEAs)^8–10^.

PEAs, which we take to include persistent anticyclonic anomalies (such as blocking events) as well as long-lived quasi-stationary cyclonic anomalies, can arise through a variety of linear and non-linear dynamical mechanisms. An important example is upper-tropospheric Rossby wave breaking (RWB) — the large-scale, non-linear overturning of potential vorticity (PV) contours near the tropopause^11^. During this process, the phase speed of the associated Rossby wave tends to slow or even retrogress^12^, often leading to long-lived (>10 day), quasi-stationary circulation patterns^13^ such as blocking events^14^. Another important example occurs along the sharp PV gradients associated with subtropical jets, which act as waveguides for linear Rossby waves with eastward group speed and near zero (i.e., quasi-stationary) phase speed^15–18^. These waveguide modes manifest in observations as circumglobal teleconnection patterns^18^. The low- and high-pressure anomalies that comprise the troughs/ridges of the wave packets can persist over a given region for extended periods of time and significantly influence local near-surface conditions^19^.

**Figure 1:**
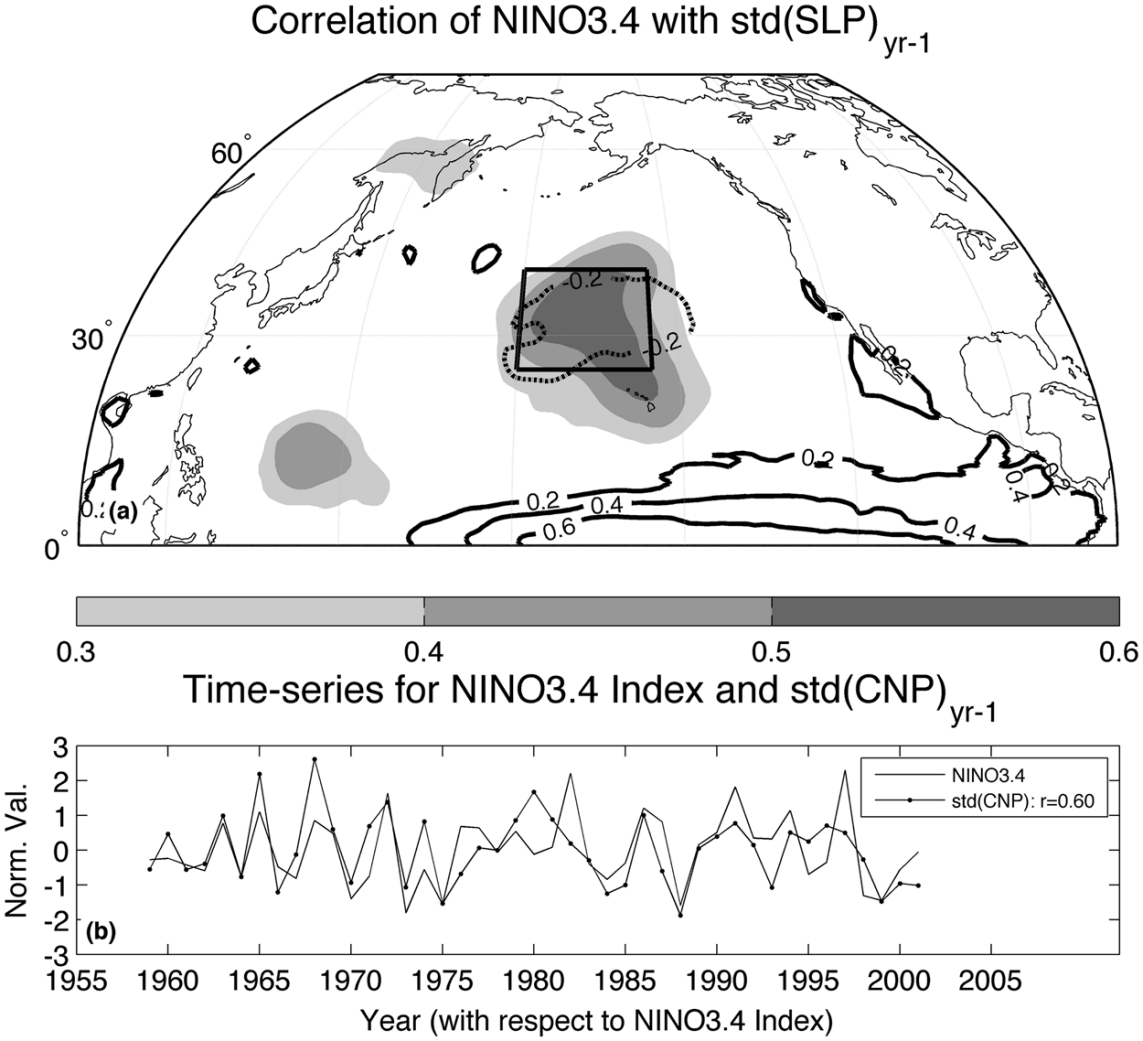
Intraseasonal extratropical atmospheric variability prior to El Nin∼o/Southern Oscillation events. (**a**) Shading: Correlation between boreal winter (Nov.-Mar.) standard deviation of daily-mean SLP and the seasonal-mean NINO3.4 index 12 months later (Dec.-Feb) for the period 1958-59 to 2000-01. Shading interval given by color bar at the bottom of the panel. Contours: regression of Dec.-Feb. sea surface temperatures (SST) against daily SLP variability in the Central North Pacific (CNP—designated by the box) during the prior extended boreal winter. Units - (K). Contour interval as labeled. (**b**) Normalized time-series of Dec.-Feb NINO3.4 index (solid line) and daily SLP variability in the CNP during the prior extended boreal winter (dotted line). Correlation between the two time-series given in the legend; the correlation value is statistically significant at the *p* < *0.01* level, based upon a two-tailed t-test. SLP data taken from the European Centre for Medium-Range Weather Forecasts (ECMWF) Reanalysis (ERA-40) for 1959–2002. SST data taken from ORA-S3 ECMWF ocean reanalysis. Daily SLP variations at a given grid-point derived by first removing from the daily values the long-term climatological mean for that day, then calculating the standard deviation of the anomalous daily SLP values about the seasonal-mean anomaly for the given year. Daily SLP variability in the CNP derived by calculating the standard deviation of the anomalous daily SLP values about the seasonal-mean anomalies for the given year and then area averaging the grid-point values within the region 20-40 N; 155-180 W. The map in this figure is generated by MATLAB R2014a using routines found in the standard Mapping Toolbox (http://www.mathworks.com/).

The large-scale, persistent, and quasi-stationary characteristics of PEAs can impart a significant signature upon the seasonal-mean atmospheric circulations in the extratropics, affecting the statistics of canonical climate variability modes such as the North Atlantic Oscillation (NAO) and the Pacific/North-American pattern (PNA) along with others^9, 10, 18, 20–23^. Here we show that intraseasonal variability in the wintertime extratropical North Pacific is also linked to seasonal-mean changes in the *tropical* Pacific 9–12 months later^24^. Indeed, there is a significant and substantial correlation between day-to-day sea level pressure (SLP) variability over the Central North Pacific (CNP) during extended boreal winter (Nov.-Mar.) and the NINO3.4 index in the following year such that enhanced (reduced) daily SLP variability during a particular winter tends to precede warm (cold) conditions in the central and eastern equatorial Pacific (Fig. 1). Below, we investigate this relation further by first analyzing the statistics of daily SLP variability over the CNP to show that a leading contributor to year-to-year changes in daily SLP variability in this region results from year-to-year changes in the frequency of low-pressure days. Next we show that the year-to-year changes in the frequency of low-pressure days impart a significant signature upon the seasonal-mean SLP and wind fields. To ascertain the source of these low-pressure events we then apply an objective algorithm to identify PEAs and demonstrate that year-to-year changes in daily SLP variability over the CNP is primarily associated with the frequency of low-pressure PEAs during a particular winter, which through their influence on seasonal-mean subtropical SLPs and trade winds explains the relation to seasonal-mean changes in the tropical Pacific 9-12 months later, as revealed in Fig. 1. We conclude by discussing wave breaking and Rossby wave trains in the extratropical North Pacific as the potential sources of the variability of PEAs.

## Results

**Figure 2:**
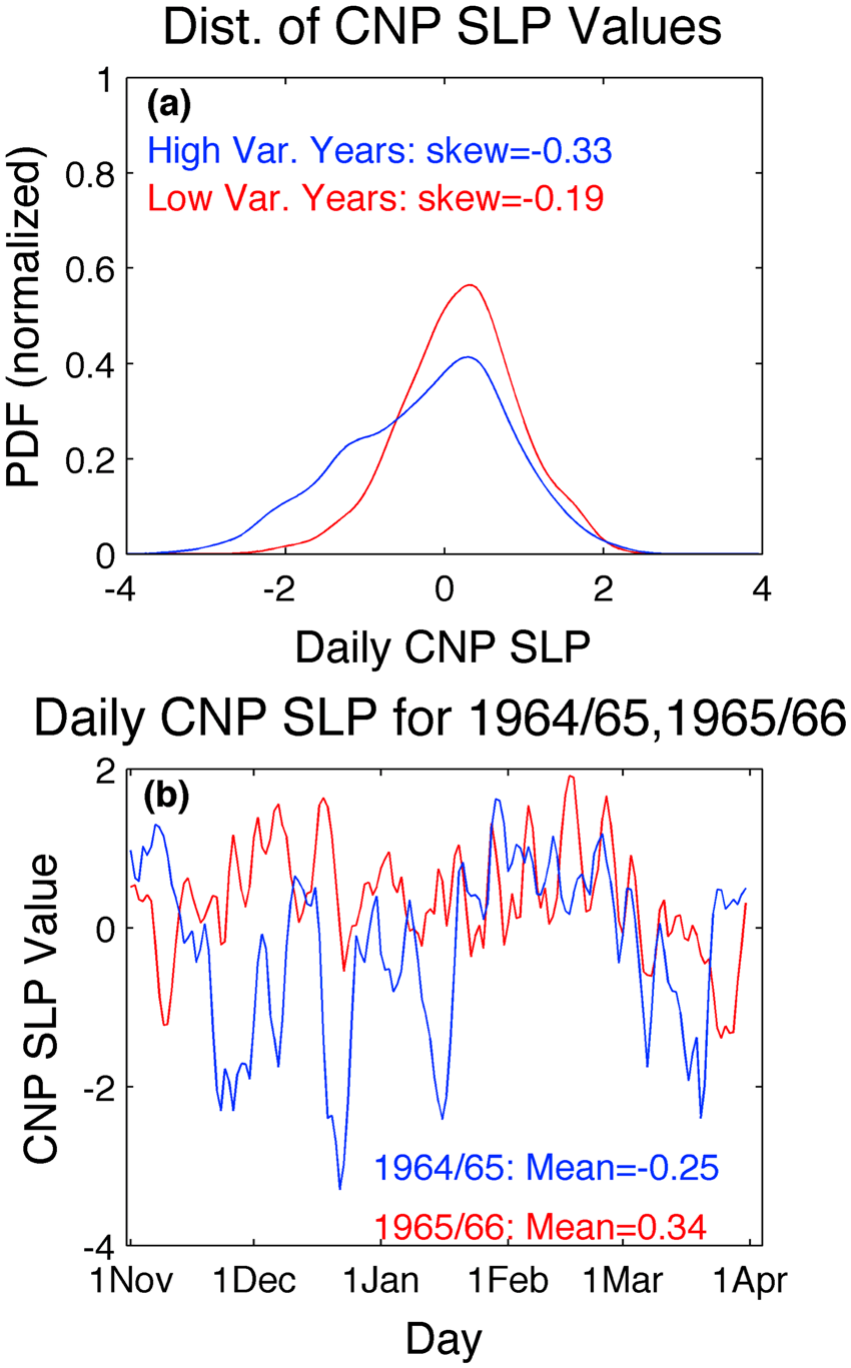
Daily distribution and evolution of sea level pressures over the central North Pacific. (**a**) Distribution of standardized daily sea level pressure (SLP) anomalies during extended boreal winter (Nov.-Mar.) averaged over the Central North Pacific (CNP), as designated by the box in Fig. 1. The distributions are calculated separately for 10 years with highest day-to-day variance in SLP (blue) and with lowest day-to-day variance in SLP (red). All distributions are centered about the mean for the given set of years and normalized by the total number of days within those years. Skewness of distributions given in the legend. (**b**) Daily evolution of standardized daily SLP anomalies averaged over the CNP during the extended boreal winters (Nov.-Mar.) of 1964-5 (blue) and 1965-66 (red). Years are chosen because they have relatively high and low day-to-day variance in CNP SLP, respectively – see Fig. 1b. Seasonal mean CNP SLP values for each year given in the legend.

Investigation of the nature of the day-to-day variability in SLP over the CNP (designated here as 20–40 N; 155–180 W) reveals that enhanced variance in the region is not symmetric about the seasonal-mean value, but is associated with increased negative skewness resulting from the occurrence of substantially more low-pressure days (Fig. 2a). Qualitatively, this can be seen by examining two characteristic winters (Fig. 2b), one with high day-to-day variability (1964-65) and one with low day-to-day variability (1965-66). As is evident, enhanced variability during the former year is manifested as frequent and persistent (>5 day) periods of lower-than-normal pressures. Importantly, though the day-to-day variability is calculated about the seasonal-mean value for the given year, there is a significant negative correlation between the standard deviation of daily-mean CNP SLP and the seasonal-mean SLP value during the given winter (*r* = −0.52; *p* < *0.01* based upon a two-tailed t-test), indicating that the preponderance (absence) of lower-than-normal pressures imparts a significant negative (positive) signature upon the seasonal-mean fields.

**Figure 3:**
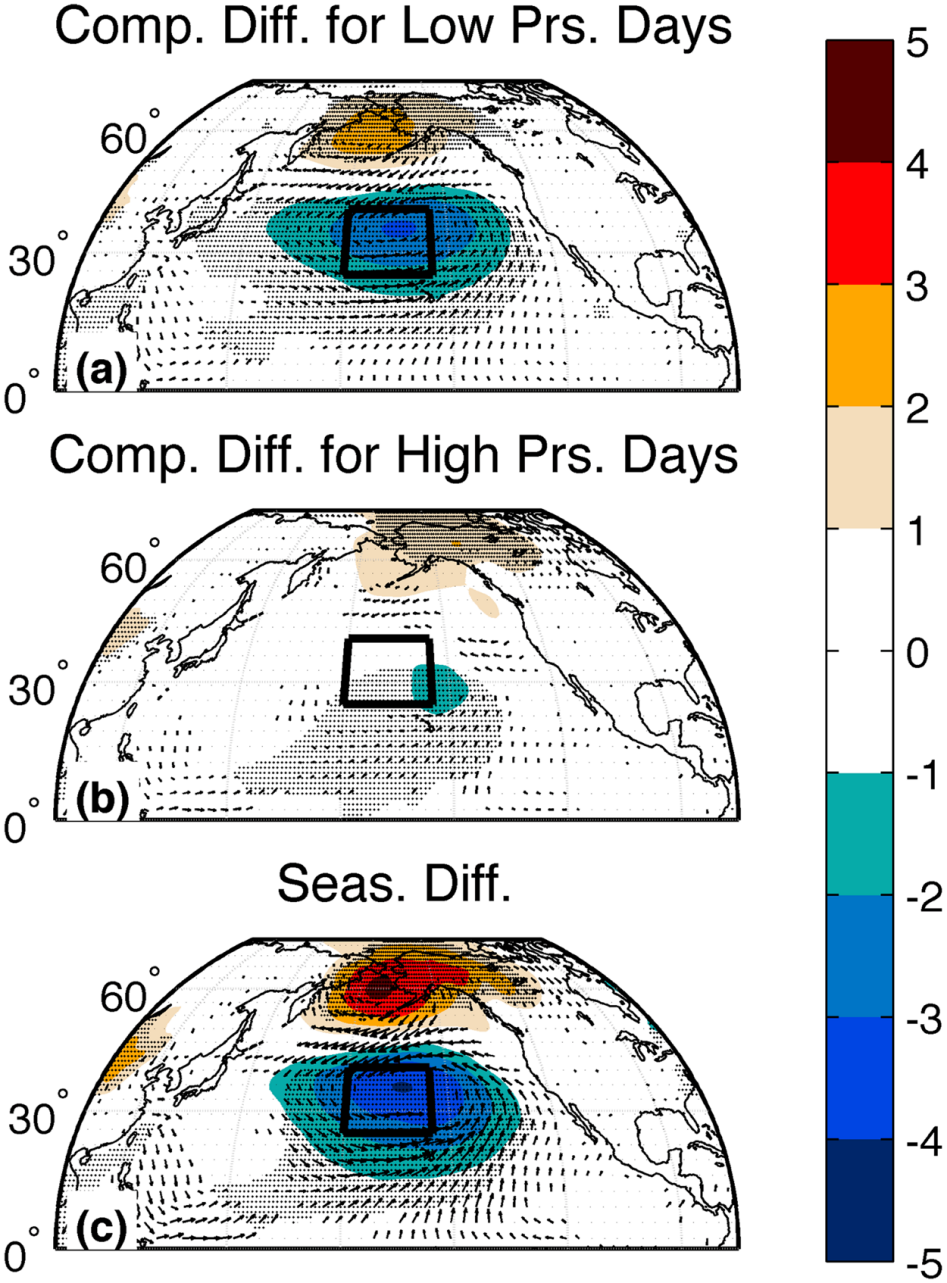
Daily central North Pacific circulation patterns during low and high variance years. **(a)** Shading: Difference in composite-mean accumulated daily sea level pressure (SLP) anomalies during extended boreal winter (Nov.-Mar.) for days in which SLP averaged over the central North Pacific (CNP) is smaller than its climatological value (see Methods). Composite-means calculated for 10 years with highest day-to-day variance in CNP SLP and 10 years with lowest day-to-day variance in CNP SLP. Composite-mean accumulated values are normalized by the number of days in the season (151). Units - (hPa). Shading interval given by color bar at the right of the figure. Hatching: Areas where the two composite-mean accumulated daily SLP anomalies are statistically significantly different from one another at the *p* < *0.1* level, based upon a two-tailed t-test. Vectors: Same as shading except for difference in composite-mean accumulated 10 m wind anomalies. Only shown are vectors in which at least one component of the composite-mean accumulated daily 10 m wind anomalies are statistically significantly different from one another at the *p*<*0.1* level, based upon a two-tailed t-test. (**b**) Same as (**a**) except for days in which SLP averaged over the CNP is greater than its climatological value. (**c**) The sum of the values in (**a**,**b**) which by construction represents (exactly) the seasonal mean difference in SLP and 10 m winds during the 10 years with highest day-to-day variance in CNP SLP and the 10 years with lowest day-to-day variance in CNP SLP. These panels indicate that years with enhanced day-to-day variance in CNP SLP experience greater accumulation of lower-than-normal SLP than years with reduced day-to-day variance in CNP SLP (panel **a**). Further, they experience less accumulation of higher-than-normal SLP (panel b), although the magnitude of this reduced accumulation is much less than in (panel **a**). As a consequence, the main contributor to the seasonal-mean reduction in CNP SLP during high-variance years (panel **c**) is increased frequency and magnitude of days with lower-than-normal SLP. The maps in this figure are generated by MATLAB R2014a using routines found in the standard Mapping Toolbox (http://www.mathworks.com/).

The influence of lower-than-normal pressure events on the seasonal-mean pressure and circulation characteristics is confirmed by the difference in the composite-mean contribution of high (low) pressure days to the seasonal-mean value during high-variance years *vis-à-vis* low-variance years (Fig. 3; see Methods). Overall, the accumulated influence of daily pressure variations on the seasonal mean value during days with relatively-low SLPs over the North Pacific shows substantially reduced (i.e. larger negative) values during high-variance years as compared with low-variance years. By contrast, the accumulated influence of daily pressure variations to the seasonal mean value during days with relatively-high SLPs over the CNP is only slightly reduced during high-variance years as compared with low-variance years, which further highlights that enhanced intraseasonal CNP SLP variability does not result from increased frequency and/or magnitude of daily high pressures (were such the case, the expectation is that the accumulated influence would be substantially higher during high-variance years). When aggregated over the full season, the seasonal-mean composite values during high-variance years show a substantial and significant reduction in SLP values over the mid-latitude North Pacific, with a corresponding increase in SLP values over the sub-polar North Pacific (note that this latter feature is found both during days with relatively low and relatively high SLP values over the central Pacific—Fig. 3a,b—and hence represents the seasonal-mean structure underlying the day-to-day variations, not a response to these variations as in the lower latitudes).

In addition to influencing the seasonal-mean pressure fields over a broad swath of the North Pacific, the preponderance/absence of lower-than-normal pressure events during a given year also influences the seasonal mean surface wind fields (Fig. 3a,c). Not only is this influence felt in the mid- and high-latitudes, but importantly extends equatorward into the tropics where it modulates the strength and structure of the trade winds and alters both the surface^25^ and sub-surface^26, 27^ temperatures of the equatorial Pacific.

**Figure 4:**
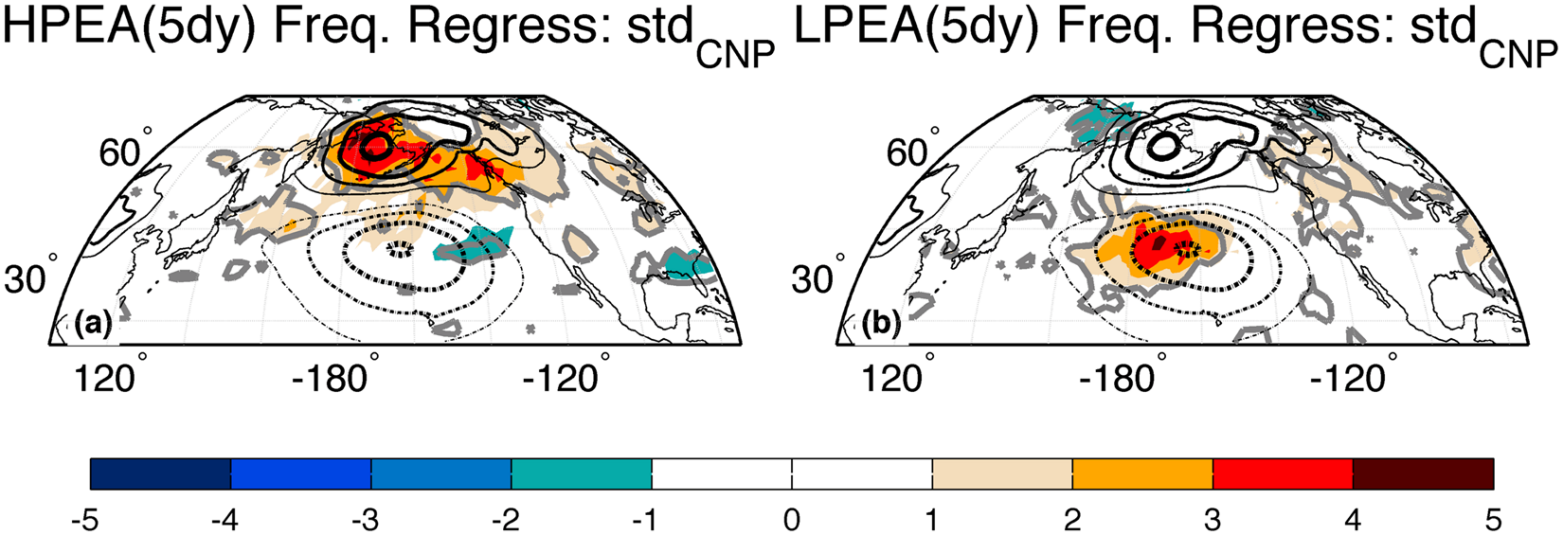
Persistent extratropical anomalies (PEAs) during years with enhanced variance in daily sea level pressures over the central North Pacific. (**a**) Shading: Number of days experiencing high-pressure persistent (>5 day) extratropical anomalies (HPEAs) during extended boreal winter (Nov.-Mar.) regressed against daily sea level pressure (SLP) variability over the central North Pacific (CNP), as represented by the time-series in Fig. 1b. Units - (days). Shading interval given by color bar at the bottom of the figure. Grey contour: Areas where the HPEA-day regression values are statistically significant at the *p* < *0.1* level, based upon a two-tailed t-test. Black contours: The seasonal mean difference in SLP during the 10 years with highest day-to-day variance in CNP SLP and the 10 years with lowest day-to-day variance in CNP SLP, as shown in Fig. 3c. Contour interval is 1hPa; positive (negative) values shown as solid (dashed) lines; the 0-contour is omitted. (**b**) Shading, Grey contour: same as (**a**) except for number of days experiencing low-pressure PEAs (LPEAs) during extended boreal winter (Nov.-Mar.). Black contours: same as (**a**). The HPEA (LPEA) statistics are calculated using a two-dimensional index that is adopted from a blocking index^36, 37^: first the seasonal cycle and interannual variability are removed from the daily 500hPa geopotential height field^38^ and then all grid points are searched for stationary positive (negative) anomalies that are larger (smaller) than one standard deviation for at least 5 days and satisfy a minimum spatial scale^36^. The maps in this figure are generated by MATLABs R2014a using routines found in the standard Mapping Toolbox (http://www.mathworks.com/).

Time-series analysis of the day-to-day CNP SLP variations reveals that enhanced variability during high-variance years is consistent with long-lived features that extend beyond synoptic (i.e. 3–5 day) timescales (Supplementary Fig. S1). Further, lead-lag maps of the composite pressure and wind field evolution highlight the quasi-stationary nature of the SLP variations over the central Pacific (Supplementary Fig. S2). Together these suggest intraseasonal variability in CNP SLP is strongly tied to the preponderance and absence of low-pressure PEAs. To confirm, we regress year-to-year variance in daily CNP SLP against the number of days with persistent (>5 day) 500hPa geopotential height anomalies at each grid-point over the North Pacific, segregating based upon the sign of the anomaly (Fig. 4). Years with enhanced day-to-day CNP SLP variability correspond to enhanced frequency of low-pressure PEAs over the CNP region, but have negligible relation to the frequency of high-pressure PEAs in the CNP region, again highlighting that enhanced variance in CNP SLP results from the preponderance/absence of transient intrusions of lower-than-normal pressures into this region. In fact, over half the interannual variance in day-to-day CNP SLP variability can be explained by variations in the frequency of persistent low-pressure PEAs over the region (r = 0.74; *p* < *0.01*, based upon a two-tailed t-test), emphasizing the importance of the long-lived, non-synoptic (i.e. >5 day) timescale low-pressure intrusions upon day-to-day variability in this region.

## Discussion

As discussed above, enhanced frequency of low-pressure PEAs over the midlatitude North Pacific during years with high SLP variance co-locates with substantial and significant reduction in seasonal-mean SLP values over the midlatitude North Pacific, with a corresponding increase in SLP values over the sub-polar North Pacific, characteristic of the negative phase of the North Pacific Oscillation (NPO)^28, 29^. Seasonal-mean changes in the NPO, and the southern lobe in particular, influence the state of the ocean and atmosphere in both the subtropics and tropics^30, 31^. While the southern lobe of the NPO is influenced in part by the meridional overturning associated with the regional Hadley cell circulation over the Pacific^32^, here we argue that the southern lobe of the NPO, along with its influence on the state of the tropical Pacific, is in part also a manifestation of the preponderance and/or absence of low-pressure PEAs during a particular winter. Indeed the strong concurrence of enhanced intraseasonal CNP SLP variability with the leading SLP-related ENSO precursor^30, 31^ (Supplementary Fig. S3) helps to explain the correspondence between variations in intraseasonal extratropical North Pacific circulations and seasonal-mean changes in the tropical Pacific 9-12 months later, as revealed in Fig. 1.

This interpretation of the NPO as at least partly a seasonal-mean signature of increased/decreased occurrence of PEA-related SLP anomalies raises the issue of determining the nature of the dynamical mechanisms underlying these changes in PEA frequency. As noted in the introduction, there are at least two important sources for PEAs. One possibility, of nonlinear nature, is that enhanced frequency of low-pressure PEAs is generated by enhanced upper-tropospheric RWB activity, as suggested elsewhere (*cf*. ref. 22 and their discussion of the West Pacific Pattern, which is the upper-tropospheric manifestation of the NPO^33^). Indeed, there is a significant relation between year-to-year changes in the variance of daily CNP SLP and the number of days with persistent (>5 day) reversals of the near-tropopause potential temperature gradient over the North Pacific (Supplementary Fig. S4). The results show more frequent persistent reversals, which indicate enhanced RWB activity^14^, during winters with high daily CNP SLP variance. The details of this relation, as well as the potential influence of different types of wave breaking events (e.g., cyclonic versus anticyclonic)^34, 35^, merit further investigation.

Another possibility, of linear nature, is that the increased frequency of low-pressure PEAs is due to enhanced Rossby wave packet activity in the Pacific subtropical jet waveguide, which leads to meridional excursions of the upper-tropospheric jet and a negative phase of the NPO (with lower-than-normal SLP values in the southern lobe^33^). Examination of the upper-tropospheric meridional winds prior to and following days with relatively-low CNP SLPs (Supplementary Fig. S5) confirms its relation with significant and substantial variations in the meridional flow at this level. Further, we note that these variations in upper-level winds show a clear eastward group speed, near zero (i.e. quasi-stationary) phase speed and a structure akin to that of wave packets known to induce cold-air outbreaks in the eastern United States^19^ that help explain the NPO-related seasonal-mean^33^ and synoptic^29^ temperature signatures in this region. Overall these preliminary analyses highlight that the enhanced intraseasonal variability in extratropical North Pacific atmospheric circulation examined here may have multiple mechanistic drivers, the nuances of which will need to be analyzed both in isolation as well as jointly.

## Methods

### Composite-mean accumulated anomalies

To estimate the contribution of daily atmospheric anomalies during high (low) CNP SLP days to the seasonal-mean value for a given year, we first recognize that the seasonal mean anomaly (for a given parameter at a given grid point) is simply the sum of the daily anomalies over the course of the season, divided by the number of days within the season:

1 $$\overline{P(yr)}=\frac{{\sum }_{d=1}^{151}P^{\prime} (d,yr)}{{\sum }_{d=1}^{151}d}$$

here $\overline{P(yr)}$ is the seasonal (mean) anomaly for the parameter, *P*, for the given year, *yr*, and $P^{\prime} (d,yr)$ is the daily departure of the parameter, *P*, from its climatological mean value for the given day, *d*, during the given year, *yr*. The sum itself can then be conditioned on the state of the CNP SLP index for each day of the season, such that:

2 $$\overline{P(yr)}=\frac{{\sum }_{CNP(d) < 0}P^{\prime} (d,yr)}{{\sum }_{d=1}^{151}d}+\frac{{\sum }_{CNP(d)\ge 0}P^{\prime} (d,yr)}{{\sum }_{d=1}^{151}d}$$

The first term on the r.h.s. of the equation is the contribution of daily atmospheric anomalies during low CNP SLP days to the seasonal anomaly for a given year and the second term is the contribution during high CNP SLP days, both of which are a function of the magnitude (i.e. intensity) of the anomalies during the given subset of days, and the number of days within each subset. Because these comprise a summation, i.e. accumulation, of daily anomalies for only a subset of days, we refer to them as the accumulated anomalies, ${A}^{CNP\ge 0}(yr)$ and ${A}^{CNP < 0}(yr)$:

3 $${A}^{CNP\ge 0}(yr)=\frac{{\sum }_{CNP(d)\ge 0}P^{\prime} (d,yr)}{{\sum }_{d=1}^{151}d}$$

4 $${A}^{CNP < 0}(yr)=\frac{{\sum }_{CNP(d) < 0}P^{\prime} (d,yr)}{{\sum }_{d=1}^{151}d}$$

Composite-mean accumulations can then be calculated for the 10 years with highest day-to-day variance in CNP SLP and 10 years with lowest day-to-day variance in CNP SLP. By construction, the composite-mean accumulations when summed equal the composite-mean seasonal anomaly, *e.g*.

5 $${\langle \bar{P}\rangle }_{high}=\langle {A}_{high}^{CNP\ge 0}\rangle +\langle {A}_{high}^{CNP < 0}\rangle $$

where ${\langle \bar{P}\rangle }_{high}$ is the composite-mean seasonal anomaly for the parameter, *P*, during the high-variance years, and $\langle {A}_{high}^{CNP\ge 0}\rangle$ and $\langle {A}_{high}^{CNP < 0}\rangle$ are the composite-mean accumulated anomalies during high and low pressure days, respectively. It follows, then, that differences between composite-mean seasonal anomalies during the high- and low-variance years can be decomposed into the contributions arising from daily atmospheric anomalies during high and low pressure days during these years as $\langle {A}_{high}^{CNP\ge 0}\rangle -\langle {A}_{low}^{CNP\ge 0}\rangle$ and $\langle {A}_{high}^{CNP < 0}\rangle -\langle {A}_{low}^{CNP < 0}\rangle$, respectively, while the sum of these two differences equals (exactly) the difference between the composite-mean seasonal anomalies during the high- and low-variance years. We note that results are quantitatively insensitive to whether the CNP SLP index for each day of the season is compared with its climatological value or the seasonal mean value for the given year.

## Electronic supplementary material

Supplementary Information
