# Supplementary material for: Persistent anomalies of the extratropical Northern Hemisphere wintertime circulation as an initiator of El Niño/Southern Oscillation events
Source: Sci Rep. 2017 Aug 31;7:10145. doi: 10.1038/s41598-017-09580-9 (PMC5578981; doi:10.1038/s41598-017-09580-9)
Supplement: Supplementary file 1 — Supplementary Information [file 41598_2017_9580_MOESM1_ESM.pdf]

Supplementary Information to: Persistent anomalies of the extratropical Northern Hemisphere wintertime circulation as an initiator of El Niño/Southern Oscillation events

Authors: Bruce T. Anderson[1*], Pedram Hassanzadeh[2], Rodrigo Caballero[3]

Affiliations:

[1]Department of Earth and Environment, Boston University, 685 Commonwealth Ave., Boston MA, 02215, USA

[2]Departments of Mechanical Engineering and Earth Science, Rice University, 6100 Main St., Houston TX, 77005

[3]Department of Meteorology and Bolin Center for Climate Research, Stockholm University, 106 91 Stockholm, Sweden

[*]Correspondence to: brucea@bu.edu.

Supplementary Information contains:

1. **SUPPLEMENTARY FIG. S1 | Periodicity of variations in daily central North Pacific pressures**
2. **SUPPLEMENTARY FIG. S2 | North Pacific circulation patterns associated with low pressure intrusions over central North Pacific**
3. **SUPPLEMENTARY FIG. S3 | Intraseasonal extratropical atmospheric variability and its relation to seasonal-mean changes in the North Pacific subtropical high**
4. **SUPPLEMENTARY FIG. S4 | Persistent reversals in the near-tropopause potential temperature gradient during years with enhanced variance in daily sea level pressures over the central North Pacific**
5. **SUPPLEMENTARY FIG. S5 | Upper tropospheric winds associated with low pressure intrusions over central North Pacific**

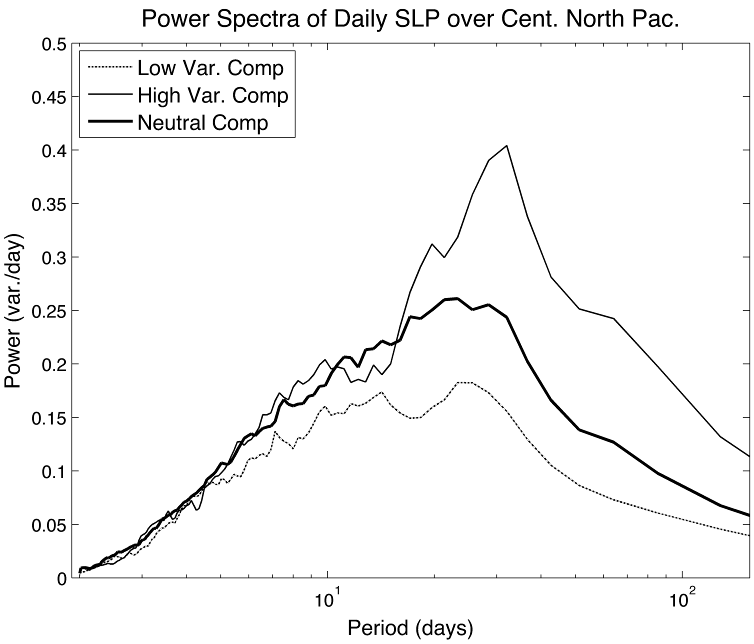

SUPPLEMENTARY FIG. S1 | Periodicity of variations in daily central North Pacific pressures. The spectral decomposition of standardized daily sea level pressure (SLP) anomalies during extended boreal winter (Nov.-Mar.) averaged over the Central North Pacific (CNP), as designated by the box in Fig. 1. The power spectra are calculated separately for the 10 years with highest day-to-day variance in CNP SLP SLP (solid), 10 years with lowest day-to-day variance in CNP SLP (dashed), and remaining 23 years with near-normal variance in CNP SLP (thick). Here, the power spectra are estimated using a multitaper method applied to the daily CNP SLP anomalies centered about the seasonal-mean anomaly for the given year and then averaged over the respective subset of years.

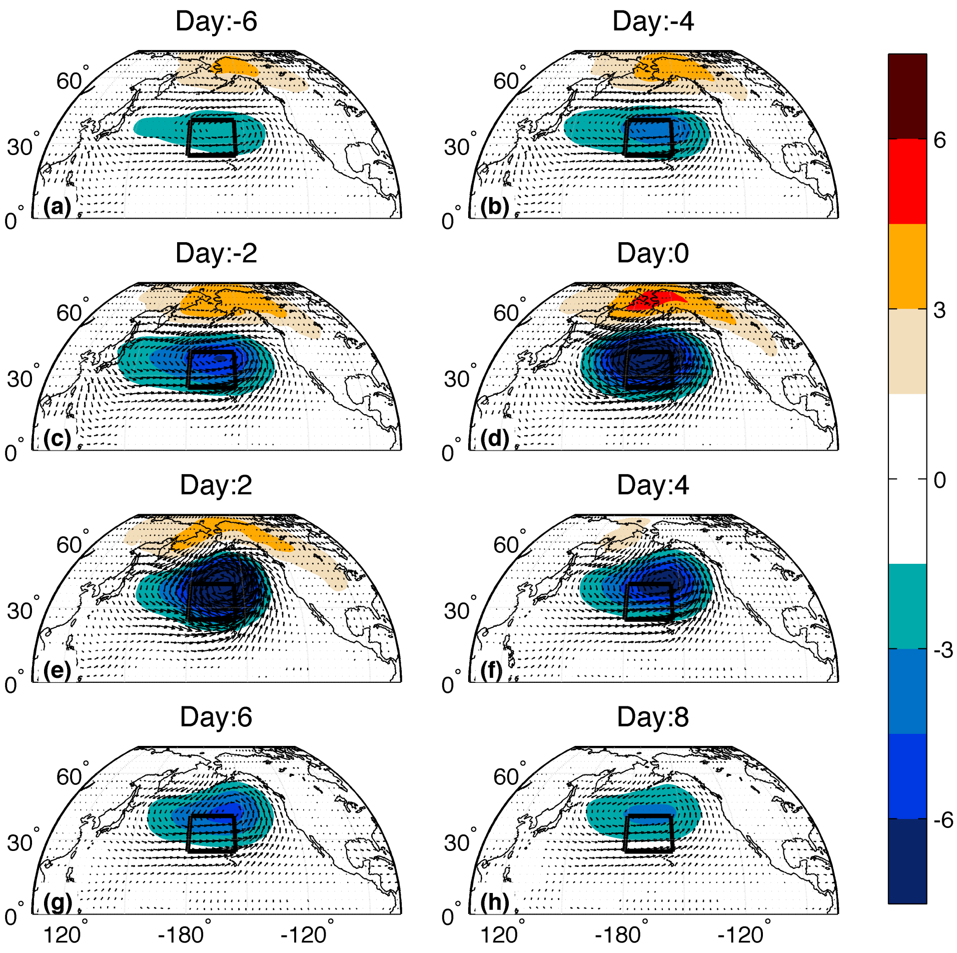

SUPPLEMENTARY FIG. S2 | North Pacific circulation patterns associated with low pressure intrusions over central North Pacific. Shading: Weighted composite-mean daily sea level pressure (SLP) anomalies during extended boreal winter (Nov.-Mar.) prior to, concurrent with, and following days in which SLP averaged over the central North Pacific (CNP) is less than its seasonal mean value. Units - (hPa). Shading interval given by color bar at the right of the figure. Weighting for composite means based upon daily value of CNP SLP on Day 0. All values shown are statistically significant at the $p<0.1$ level, based upon 1000 random replacements of each season's daily CNP SLP time-series, thereby retaining both the autocorrelation and variance of the predictor and predictand fields. Vectors: Same as shading except for weighted composite-mean 10m wind anomalies. Only shown are vectors in which at least one component of the weighted composite-mean daily 10m wind anomalies are statistically significant at the $p<0.1$ level, based upon 1000 random replacements of each season's daily CNP SLP time-series. **a-h**, daily weighted composite-mean fields prior to (negative lead/lag values), concurrent with (Day 0) and following (positive lead/lag values) days with low-pressure intrusions over the CNP. The maps in this figure are generated by MATLAB R2014a using routines found in the standard Mapping Toolbox (http://www.mathworks.com/).

 136

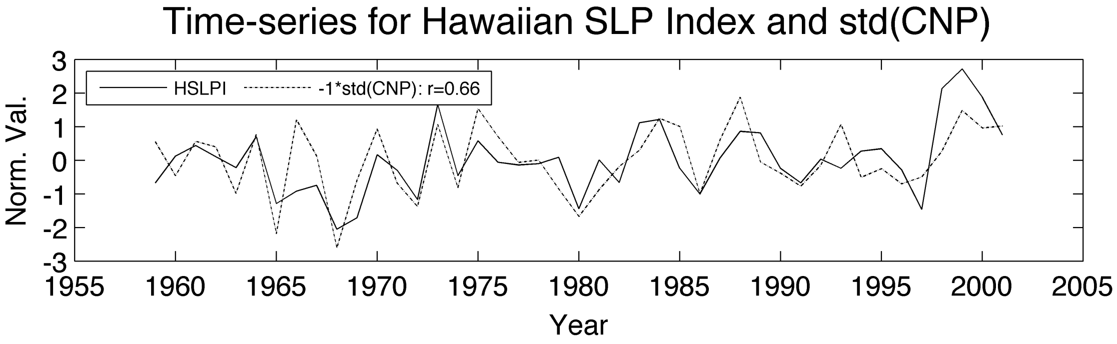

SUPPLEMENTARY FIG. S3 | Intraseasonal extratropical atmospheric variability and its relation to seasonal-mean changes in the North Pacific subtropical high. Time-series of daily sea level pressure (SLP) variability over the central North Pacific (CNP) during extended boreal winter (Nov.-Mar.), as represented by the time-series in Fig. 1b (dashed line), and of seasonal-mean SLP anomalies over the subtropical North Pacific, as represented by the Hawaiian SLP Index during the concurrent extended boreal winter (HSLPI – solid line). The HSLPI—the leading SLP-related precursor to ENSO events the following winter[31]—is derived by first removing from the seasonal-mean values the long-term climatological mean then normalizing by the standard deviation of the anomalous seasonal-mean SLP values across all years. The normalized seasonal-mean anomalies are then area averaged within the region 10-25N; 175-140W.

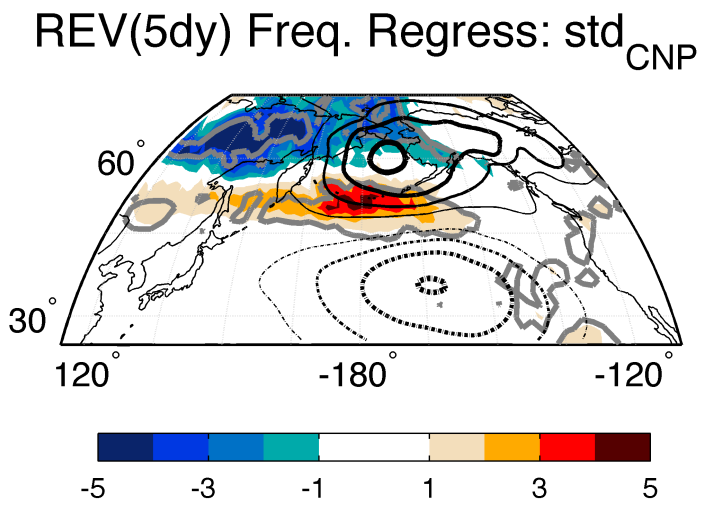

SUPPLEMENTARY FIG. S4 | Persistent reversals in the near-tropopause potential temperature gradient during years with enhanced variance in daily sea level pressures over the central North Pacific. Shading: Number of days experiencing persistent (>5day) reversals (REV) of the potential temperature gradient on the dynamical tropopause during extended boreal winter (Nov.-Mar.) regressed against daily sea level pressure (SLP) variability over the central North Pacific (CNP), as represented by the time-series in Fig. 1b. Units - (days). Shading interval given by color bar at the bottom of the figure. Grey contour: Areas where the REV-day regression values are statistically significant at the $p<0.1$ level, based upon a two-tailed t-test. Black contours: The seasonal mean difference in SLP during the 10 years with highest day-to-day variance in CNP SLP and the 10 years with lowest day-to-day variance in CNP SLP, as shown in Fig. 3c. Contour interval is 1hPa; positive (negative) values shown as solid (dashed) lines; the 0-contour is omitted. Dynamical tropopause is defined as where potential vorticity (PV) equals 2 PV units[14]. The REV statistics are obtained using a two-dimensional index: first, the daily large-scale meridional temperature gradient on PV=2 contour is calculated using Eq. (4) of Ref. 14 around every grid point between 20N and 80N; second, grid points with positive gradients for at least 5 days are identified. The maps in this figure are generated by MATLAB R2014a using routines found in the standard Mapping Toolbox (http://www.mathworks.com/).

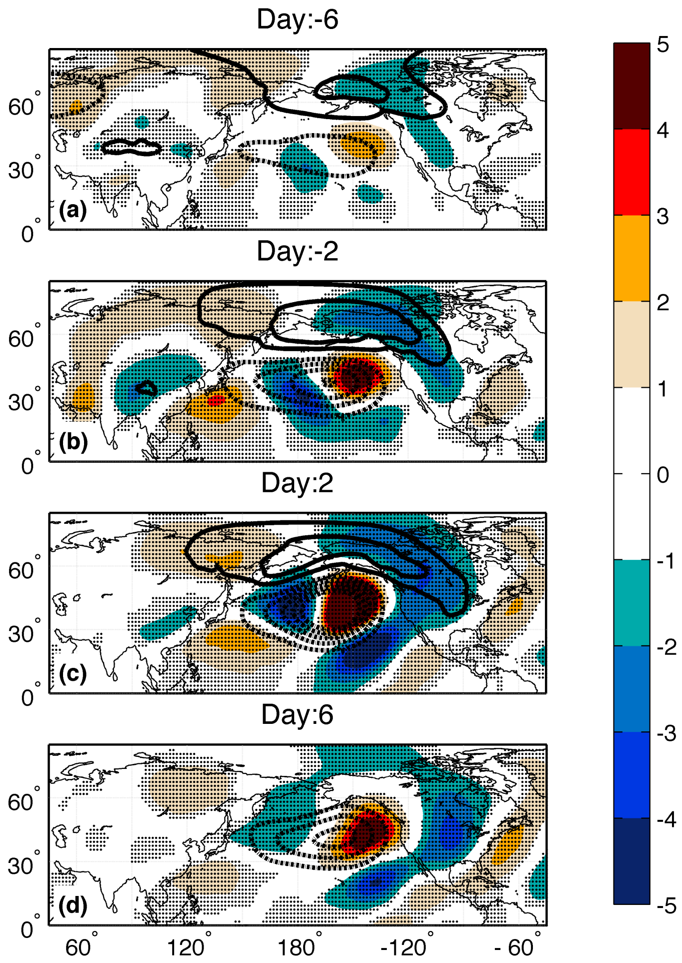

SUPPLEMENTARY FIG. S5 | Upper tropospheric winds associated with low pressure intrusions over central North Pacific. Shading: Weighted composite-mean daily 250 hPa meridional wind anomalies during extended boreal winter (Nov.-Mar.) prior to and following days in which sea level pressure (SLP) averaged over the central North Pacific (CNP) is less than its seasonal mean value. Units - (m/s). Shading interval given by color bar at the right of the figure. Weighting for composite means based upon daily value of CNP SLP on Day 0. Hatching: Areas where the weighted composite-mean daily wind anomalies are statistically significant at the $p<0.1$ level, based upon 1000 random replacements of each season's daily CNP SLP time-series, thereby retaining both the autocorrelation and variance of the predictor and predictand fields. Contours: Same as shading except for weighted composite-mean SLP anomalies. Units - (hPa). Values and contour interval same as in Supplementary Fig. S2. Solid (dashed) lines represent positive (negative) values. 0-contour value is omitted for clarity. **a-d**, daily weighted composite-mean fields prior to (negative lead/lag values) and following (positive lead/lag values) days with low-pressure intrusions over the CNP. The maps in this figure are generated by MATLAB R2014a using routines found in the standard Mapping Toolbox (http://www.mathworks.com/).
